# Enhancing residents’ neonatal resuscitation competency through team-based simulation training: an intervention educational study

**DOI:** 10.1186/s12909-023-04704-4

**Published:** 2023-10-10

**Authors:** Roya Farhadi, Bita Khalili Azandehi, Fattane Amuei, Mozhgan Ahmadi, Atefeh Zabihi Zazoly, Ali Asghar Ghorbani

**Affiliations:** 1 Associate Professor, Pediatric Infectious Diseases Research Center, Mazandaran University of Medical Sciences, Sari, Iran; 2 Ph.D. of Medical Education, Social Security Organization, Education & Research Unit, Valiasr Regional Hospital, Sari Azad university, Ghaemshahr, Iran; 3 Assistant Proffessor, Center for Studies and Development of Medical Sciences Education, Mazandaran University of Medical Sciences, Sari, Iran; 4 Head Nurse of Neonatology ward, Boo-Ali Sina educational and therapeutic center, Mazandaran University of Medical Sciences, Sari, Iran; 5 Assistant Professor, Operating Room Department, School of Allied Medical Sciences, Mazandaran University of Medical Sciences, Sari, Iran; 6 Assistant professor, School of Aliied Medical Sciences, Mazandaran University of Medical Sciences, Sari, Iran

**Keywords:** Newborn, Resuscitation, Simulation, Education, Teamwork, Debriefing

## Abstract

**Background:**

Neonatal resuscitation training in a simulated delivery room environment is a new paradigm in pediatric medical education. The purpose of this research is to highlight team-based simulation as an effective method of teaching neonatal resuscitation to senior pediatric residents.

**Methods:**

In an intervention educational study, we evaluated the impact of team-based simulation training in the development of neonatal resuscitation. A team consisting of a three-person group of senior pediatric residents performed neonatal resuscitation on a low-fidelity newborn simulator based on the stated scenario. Video-based structured debriefing was performed and followed by the second cycle of scenario and debriefing to evaluate the feasibility of conducting team-based simulation training in a lesser-resourced environment. Evaluation criteria included megacode scores which is a simulation performance checklist, pre-and post-test scores to evaluate residents’ knowledge and confidence, the survey checklist as a previously developed questionnaire assessing residents’ satisfaction, and debriefing from live and videotaped performances. Four months after the end of the training course, we measured the behavioral changes of the residents by conducting an OSCE test to evaluate post-training knowledge retention. Mean ± SD was calculated for megacode, satisfaction (survey checklist), and OSCE scores. Pre- and post-program gains were statistically compared. The first three levels of Kirkpatrick’s training effectiveness model were used to evaluate the progress of the program.

**Results:**

Twenty-one senior residents participated in the team-based simulation. The mean ± SD of the megacode score was 35.6 ± 2.2. The mean ± SD of the overall satisfaction score for the evaluation of the first level of the Kirkpatrick model was 96.3 ± 3.7. For the evaluation of the second level of the Kirkpatrick model, the pre-posttest gain in overall confidence score had a statistically significant difference (P = 0.001). All residents obtained a passing grade in OSCE as an evaluation of the third level.

**Conclusions:**

Team-based simulation training in neonatal resuscitation improves the knowledge, skills, and performance of pediatric residents and has a positive effect on their self-confidence and leadership skills. There is still a need to investigate the transfer of learning and abilities to real-life practice, and further research on cost-effectiveness and impact on patient outcomes is warranted.

**Supplementary Information:**

The online version contains supplementary material available at 10.1186/s12909-023-04704-4.

## Introduction

Approximately 10% of all newborns need some basic levels of resuscitation at birth, and less than 1% require advanced steps [1, 2]. Therefore, well-trained neonatal resuscitation providers must be available if such measures are needed [1]. Neonatal resuscitation program (NRP) guidelines have been developed by the American Academy of Pediatrics (AAP), International Liaison Committee on Pediatrics (ILCOR), and American Heart Association(AHA) to provide life-saving care to improve birth outcomes when emergencies occur [3].

Before 2010, the NRP program focused on acquiring knowledge and technical skills related to neonatal resuscitation. The sixth edition of the NRP (2010) changed from instructor-driven training and skill stations to simulation-based learning that mimics real-life situations. The seventh edition (2016) focused more on team communication and behaviors [4]. Although simulation-based neonatal resuscitation training is now very common in developed countries, in areas with fewer resources, the traditional method of training, which is a theoretical explanation combined with a multimedia presentation, may only be applicable [1, 5]. Simulation reinforces real-life experiences with guided experiences that evoke or replicate key aspects of the real world in a fully interactive way, and simulation-based training combines theoretical knowledge and practical skills in a safe and secure environment for physicians [1, 6]. Team communication and team behaviors significantly affect the quality of neonatal resuscitation and most of the medical errors associated with increased perinatal complications and mortality are due to inappropriate teamwork [6]. Therefore, team-based training is recommended in perinatal and neonatal wards where healthcare providers perform a variety of tasks [7]. Simulation and debriefing are considered essential components, with a strong emphasis on teamwork in a simulated environment [1]. Because neonatal resuscitation scenarios are often complex and challenging, they require excellent cognitive skills, critical thinking, practical procedures, and communication skills [8]. Therefore, the old-fashioned model of apprenticeship is not recommended for pediatric residents to learn neonatal resuscitation [8].

Completing neonatal intensive care unit rotation and NRP courses during the residency program usually does not adequately prepare pediatric residents to lead neonatal resuscitation teams [9]. In addition, with the increased presence of neonatal nurses and other health professionals and the decline in patient volume in some hospitals, pediatric residents face limited opportunities to gain resuscitation experience [2]. Similarly, during this relatively short rotation, they may be less likely to expose to critically ill neonates in the delivery room [9]. The main responsibility of the pediatric residents is effective participation as a member of the neonatal resuscitation team, which focuses specifically on critically ill newborns in the first minutes after birth but, the limited time of this course to learn a large amount of knowledge may not create this opportunity in the real environment to a suitable extent [10, 11].

Pediatric residents in Iran, during the three-year study period, complete a pediatric training course with a maximum of 4 months in the neonatal intensive care unit. Therefore, low-fidelity simulation-based training may be a valuable tool for improving resuscitation skills in pediatric residents in resource-limited settings [12].

The scenarios designed in team training, due to the combination with debriefing, improve individual and team communication compared to the standard megacode which is only based on individual tests, and also the time required for team scenarios is less, which improves immediate performance [13]. Some experts recommend that each service in healthcare facilities should develop and test its core teamwork competency classification to develop teamwork training [7]].

No studies were available from developing countries, and further research has been recommended in previous studies to detect differences in neonatal resuscitation teamwork outcomes [4, 7]. Compared to other developed countries where team-based simulation is routinely performed, training pediatric residents in Iran to resuscitate neonates as a team seems challenging and different. Therefore, this study was conducted to answer our question about the role of team-based simulation training in improving the performance of residents during neonatal resuscitation in a low-resource setting.

## Materials and methods

We designed an intervention educational study and analyzed the effect of neonatal resuscitation team-based simulation on the success rate of NRP skills and the level of satisfaction of pediatric residents with this teaching method at Mazandaran University of Medical Sciences in northern Iran.

Participants included all senior pediatric residents (in the second and third years of the residency period) who rotated at Boo Ali Sina Hospital in Sari, Iran from February 2022 to May 2022. Before the initiation of the study, residents were fully informed about the intervention method and the project.

### Sample size

The sample size was estimated to be appropriate based on previous similar studies [14] and using a two-tailed alpha of 0.05, 90% power to detect a one-point difference on the pre/posttest scale with an estimated standard deviation of 1, based on responses obtained during our pilot field test. The required sample size of 23 was obtained for a study group. We were able to conduct this study with a total of 21 senior residents in our center.

### Training intervention method

Before the start of the project, due to the COVID-19 status at the start of the study, all project objectives, new updates to NRP 2021, and the goal of team-based simulation training for all residents were described in an online class. Two weeks later, with the improvement of COVID-19 in the region, a workshop on neonatal resuscitation using manikins, multimedia, and theoretical methods was held to maintain and update the information of pediatric residents about the latest version (eighth edition) of NRP 2021. All residents were assessed during an NRP advanced megacode before entering the team-based simulation project as a provider. The skills of each resident individually in evaluation, decision-making, and performance while observed by a single instructor (attending neonatologist) were scored on a megacode checklist. The checklist was guided by parameters established by the NRP Steering Committee (NRPSC) of the American Academy of Pediatrics [15] and had 19 items from 6 NRP lessons and was evaluated and completed by an observer within 5 to 10 min (Supplementary material.1). The maximum score was 38. According to this checklist, the resident must obtain at least 85% of the total scores for the various items by the end of the scenario designed by the instructor.

Residents who were not able to pass the advanced standard megacode test were excluded from the study and entered the project in the next stages after obtaining a passing score. Finally, qualified residents in three-person groups entered the team-based simulation study. A part of the Neonatal Intensive Care Unit (NICU) was set up to maintain the educational environment. Each group arrived at the area according to the schedule to receive their scenario. The resuscitation scenario was announced to the team by an attending neonatologist who was NRP certified. Residents were given an introductory stem. The first scenario involved the resuscitation of a premature infant born to a mother with a history of severe preeclampsia and the second scenario involved a term newborn with prenatal asphyxia and fetal distress. Both scenarios described apneic newborns with persistent cyanosis and bradycardia for which residents should perform initial steps, mask ventilation, corrective steps, intubation, chest compression, and administration of epinephrine via the endotracheal tube or a simulated umbilical venous catheter line. The scenarios were designed to cover all key aspects of neonatal resuscitation. If residents were unable to proceed, they were redirected by the instructor. The learning objectives of the scenarios included conducting a team briefing, recognizing the indications for and performing effectively the steps of neonatal resuscitation. The team leader (who was chosen by the residents themselves), assigned the roles to the other team members and made appropriate treatment decisions based on the newborn’s signs and symptoms as announced by the attending neonatologist. Resuscitation was started by the team and continued for up to 10 min and stopped whenever the scenario reached the moment when the newborn began to breathe, his heart rate exceeded 100 beats per minute, and the oxygen saturation was reported acceptable.

A staff nurse participated as an actor to reflect on a real-life situation. We used the Health Baby Breath (HBB) manikin (Laerdal Medical, in Stavanger, Norway) to mimic a newborn baby. During this time, the staff nurse recorded the video of the resuscitation scenario focusing on the baby and only the hands of the team members (supplementary material 2). After the end of the scenario, the residents reviewed the recorded video, and immediately a structured debriefing session was conducted in the same environment between the team residents, and the attending neonatologist, for 10–15 min. A debriefing strategy with a plus-delta approach was used to identify what went well and what needed to be changed. The scenario debriefing cycle was repeated with a second scenario requiring residents to perform advanced neonatal resuscitation procedures including intubation. Scenarios were adopted from the eighth edition of the NRP manual, and team members were required to perform the NRP algorithm step by step to perform a successful resuscitation. The simulation scenario and the training sessions concluded with an emphasis on key points for enhancing future performance and feedback was given to them regarding strengths and weak points. During the debriefing, the attending neonatologist/facilitator encouraged the residents to reflect on what went well and what could be improved. Although debriefing occurred in the simulation setting, the video recording was done to achieve accurate recall.

### Training evaluation method

Pre-test and post-test were taken from residents before and after the project. The pre-test and post-test were conducted in the form of a multiple-choice test (MCQ) based on the questions at the end of each lesson in the 8th edition neonatal resuscitation textbook(15), with a total of 20 points to determine the knowledge, attitude, and decision to practice of the residents. The tests evaluated main skills including initial steps, positive pressure ventilation, chest compression, and drug administration. Then, to measure the desirability of the course, a survey form (with 20 items) was created regarding the content, instructor, and organization of the course in the form of an anonymous questionnaire using a 5-point Likert scale (weak = 1, average = 2, good = 3, very good = 4, excellent = 5) [Supplementary material 3].On the other hand, trainees also provided specific feedback and suggested specific strategies for practice to increase knowledge, behaviors, and skills. The first three levels of Kirkpatrick’s training effectiveness model were used to evaluate the progress of residents [16]. To evaluate level one (reaction), the sum of the points of the survey form filled by the residents was calculated as the overall satisfaction score. The pre and post-test were conducted to evaluate the second level (learning). Furthermore, the observation method by the study investigator (NRP-certified attending neonatologist) and video-based debriefing was used.

For the evaluation of the third level (behavior), four months after the end of the training course, we measured the behavioral changes of the residents by conducting an Objective Structured Clinical Examination (OSCE) test to ensure whether the learners understood what they learned in the course and used it while doing their work or not. The examination consisted of five “stations”, lasting 25 min. In station 1, the resident’s briefing ability knowledge was assessed. In stations 2–5, the resident’s skills in the initial steps of resuscitation, positive pressure ventilation, intubation/ chest compression, and medication were assessed. Time management and residents’ re-evaluation of the neonate were also assessed. The test was graded from 0 to 10, and a minimum score of 5 was required to pass. The scenario was designed by the attending neonatologist and the implementation process was supervised by her.

### Data analysis

Descriptive statistics were used to analyze the data using SPSS version 26. Means and standard deviations were calculated for megacode scores, overall satisfaction scores (from survey form), and OSCE scores. The means of answers to the pre-and post-tests were analyzed using the Wilcoxon signed-rank test. The percentage of residents who passed the OSCE exam was calculated. P-value < 0.05 was considered significant.

## Results

Twenty-one senior residents participated in the neonatal resuscitation team-based simulation. Thirteen of them (61.9%) were female.13 (62%) residents were in the second year and 8(18%) were in the third year. All residents were assessed during an advanced megacode. Eighteen of them passed it in the first session and three residents could not obtain the minimum score (32 out of 38) on the first time and passed it after receiving a booster session based on skills missed in the initial implementation. The mean ± standard deviation of the megacode score was 35.6 ± 2.2 and the minimum and maximum scores were 32 and 38, respectively. The details of the scores obtained by the residents for each task and the percentage of residents who did each task correctly in order are given in Table 1.

**Table 1:** Scores of Neonatal Resuscitation Program megacode assessment form and the percentage of residents who performed each task correctly in order

Category	Lesson	Mean ± SD	Done correctly in order (%)
Team briefing (maximum point = 8)		7.5 ± 0.6	
Asks 4 pre-birth questions (Expected GA, Fluid clear, UC management plan, Risk factors)	2	2 ± 0	100
Discusses plan and assigns roles to team members	2	2 ± 0	100
Checks Equipment inc. Bag, Mask & Oxygen Supply	2	1.8 ± 0.3	88.8
Asks 3 assessment questions (Term, Tone, Breathing or Crying)	2	1.7 ± 0.4	72.2
Initial steps of resuscitation (maximum point = 4)		3.5 ± 0.6	
Warm dry stimulate and removes wet towels Position the airway and suction if necessary	3	1.7 ± 0.4	72.2
Assesses respirations +/- heart rate Initiates monitoring for pulse oximeter probe to right wrist	3	1.8 ± 0.2	94.4
Positive-pressure ventilation (maximum point = 8)		7.6 ± 0.4	
Indicates need for and initiates positive positive-pressure ventilation(Apnea or gasping, heart rate)	4	1.9 ± 0.2	94.4
Checks for rising heart rate after 15 s of PPV	4	1.9 ± 0.2	94.4
Takes corrective action when heart rate not rising & chest not moving (Mask readjustment Reposition; Suction mouth & nose Open mouth; Pressure increase; Alternate airway)	4	1.8 ± 0.4	77.7
Provides effective positive pressure ventilation (40–60 bpm) for 30 s	4	1.8 ± 0.3	88.8
Chest compression (maximum point = 8)		7.5 ± 0.6	
Re-evaluates heart rate(Heart rate < 60 bpm) Consider intubation and apply ECG if not already done	4	1.8 ± 0.3	88.8
Demonstrates correct technique for intubation or assisting with intubation	5	1.9 ± 0.2	94.4
Identifies need to start chest compressions (Increases oxygen to 100%, Demonstrates correct compression technique for 60 s (2-thumb method, compression depth 1/3 anterior-posterior diameter, complete recoil of chest)	6	2 ± 0	100
Takes corrective action when heart rate not rising using the “CARDIO” mnemonic	6	1.6 ± 0.4	77.7
Medication (maximum point = 8)		7.6 ± 0.6	
Identifies need for epinephrine (Heart rate < 60 bpm)	7	1.8 ± 0.3	83.3
Identifies correct dose and route for epinephrine	7	1.8 ± 0.3	88.8
Administers ET dose while umbilical catheter being	7	2 ± 0	100
Identifies need for volume administration and administers correct solution, volume and rate of infusion	7	1.8 ± 0.3	88.8
Administers blended oxygen (maximum point = 2)		1.9 ± 0.2	
Administers blended oxygen to meet targeted saturations	3	1.9 ± 0.2	94.4
Full score for overall assessment (maximum point = 38)	2–7	35.6 ± 2.2	

The mean and standard deviation of the overall satisfaction score and the scores related to the content, instructor, and organization, for the evaluation of the first level of the Kirkpatrick model, are shown in Table 2. The residents gave an overall satisfaction score of 96 out of 100 to the educational method. The lowest satisfaction score was related to program organization and educational facilities and aids.

**Table 2:** Survey form scores to evaluate the post-simulation reaction of residents and measure the desirability of the course

Evaluation criteria	Mean ± SD	Maximum score	Minimum score
**A- Content**	29.4 ± 0.8	30	27
Applicability of the content (expression of evidence and practical scenarios)	5 ± 0	5	5
Content up-to-date	5 ± 0	5	5
Increasing your information about the training course	5 ± 0	5	5
The potential created by this session for job activities	4.4 ± 0.3	5	3
Suitability of program content to your expectations	5 ± 0	5	5
The attractiveness of the course for continued attendance	5 ± 0	5	5
**B-Professor (Instructor)**	48.7 ± 1	50	45
The ability to express and convey content	5 ± 0	5	5
Using new methods of teaching and learning	5 ± 0	5	5
The level of expertise and mastery of the instructor	5 ± 0	5	5
Presenting, progressing, and redirecting the scenario	5 ± 0	5	5
Introduction and presentation of teaching references	4.7 ± 0.4	5	4
Creating motivation and attracting the participation of learners	5 ± 0	5	5
Use of teaching instrument	4.7 ± 0.4	5	4
Create interest in asking and answering	5 ± 0	5	5
Ability to manage time	4.4 ± 0.3	5	3
General evaluation of training course management	4.7 ± 0.4	5	4
** C. Organization**	18.1 ± 2	20	14
Course notification and planning coordination	4.8 ± 0.3	5	4
Educational facilities and aids	3.5 ± 0.5	5	2
The behavior of the presenters	5 ± 0	5	5
Educational environment in terms of physical facilities	4.7 ± 0.4	5	3
Total scores (overall satisfaction score)	96.3 ± 3.7	100	89

**Table 3:** Pre and post-simulation training assessment of residents (multiple choice questionnaire scores)

Category of skills	Pre-test (mean ± SD)	Percentage of residents with correct answers(%)	Post-test (mean ± SD)	Percentage of residents with correct answers	P-value
Team briefing (maximum point = 20)	4.7 ± 8.7	25	12.7 ± 10.2	58.3	0.001
Initial steps of resuscitation (maximum point = 20)	18.1 ± 5.7	91.6	18.3 ± 5	92	0.61
Positive pressure ventilation (maximum point = 20)	15 ± 9	75	20 ± 0	100	0.001
Chest compression (maximum point = 20)	10 ± 10.4	50	18 ± 5.9	91.6	0.001
Medication (maximum point = 20)	14.6 ± 7.7	16.6	17.2 ± 8.7	83.3	0.001
Overall score (maximum point = 100)	62.6 ± 13.7	51.6	86.3 ± 9.5	85	0.001

To evaluate the second level, pre- to post-simulation training assessments, as evaluated by residents’ responses on pre- and post-training multiple choice questioners showed a positive gain and a statistically significant change (Table 3). Although there was no significant difference between the pre-test and the post-test in the scores related to the initial steps of resuscitation, the scores of other skills and the overall score of the post-test were significantly higher than the pre-test (P = 0.001). The largest mean difference was observed in the scores of the chest compression and the team briefing categories between the pre- and post-test.

In the evaluation of the third level (behavior) four months after the end of the training course, the mean score of residents’ skills using OSCE, was 8.6 ± 1.1 with minimum and maximum scores of 7 and 10, respectively. All residents obtained a passing grade and the changes were retained. On average, residents obtained 89%, 97%, 87%, 78%, and 79% of the total score in the briefing knowledge, initial steps of resuscitation, positive pressure ventilation, intubation/chest compression, and medication, respectively. Table 4 shows the details of the scores and the percentage obtained from the maximum point of each station by the residents. In total, the residents managed to achieve 86% of the maximum points of the OSCE test (10 out of 10).

**Table 4:** Objective Structured Clinical Examination (OSCE) results to assess residents’ knowledge, skills, performance, and retention of gain scores

Stations	Mean ± SD	Minimum	Maximum	Percentage of total scores which obtained by residents (%)
Team Briefing (maximum point = 2)	1.7 ± 0.2	1.6	2	89
Initial steps of resuscitation (maximum point = 2)	1.9 ± 0.1	1.7	2	97
Positive-pressure ventilation (maximum point = 2)	1.7 ± 0.3	1.4	2	87
Intubation/chest compression (maximum point = 2)	1.5 ± 0.4	1	2	78
Medication (maximum point = 2)	1.5 ± 0.4	1.2	2	79
Overall score (maximum point = 10)	8.6 ± 1.1	7	10	86

## Discussion

In this research, we evaluated the knowledge and practice of senior residents in neonatal resuscitation with the team-based simulation method, and the results showed that this program is logistically applicable and has a positive effect on the residents’ self-confidence and behavioral skills to the performance of neonatal resuscitation per guideline, following the steps correctly in appropriate timing in coordination with the team. A few studies have addressed how residents view the essential issues of teamwork during neonatal resuscitation(7). Kalaniti et al. evaluated the effect of simulation-based team training for neonatal resuscitation in senior pediatric trainees and concluded that this model of training was associated with improved learning skills regardless of whether they observed or participated(9). Residents’ primary responsibility is to participate in resuscitation with the neonatal team, specifically in the first few minutes of birth. In fact, after acquiring knowledge and skills in the junior residency course, more emphasis should be placed on teamwork and communication strategies. This training program is also accompanied by strengthening the skill of the resident to lead the resuscitation team in the real delivery room [17].

To the best of our knowledge, this is the first study that examines neonatal resuscitation based on teamwork simulation in Iran. Our study showed that it is possible to establish frequent in-situ training even in a hospital with limited resources. We used NeoNatalie, a newborn simulator designed initially to accomplish Helping Baby Breath training in low-resource settings. Indeed, the positive point of our study was that our research was conducted in a developing country where advanced simulators were not available, but we tried to use the limited facilities for neonatal resuscitation learning objectives for residents as a team-based simulation. Although some studies showed the increased effectiveness of high-fidelity simulators as a means of providing feedback and a range of difficulty levels, the low-cost newborn simulator was effective in other studies(18). High-fidelity simulators are computer-driven manikin that use pharmacological and physiological modeling algorithms to mimic real-life situations. These manikins are not only close in size to term and preterm infants, but also mimic a real airway, vital signs, and skin color (5, 19, 20). However, simulator-based training with low-fidelity manikins has more important advantages and other specific training content, such as scenarios designed by experienced facilitators and providing debriefing(5). On the other hand, previous research has shown that although high-fidelity simulator-based neonatal resuscitation training is effective on short-term outcomes, it has small to moderate benefits compared to low-fidelity simulation training(1, 5). Principles show that debriefing is an important step to determine the effectiveness of simulation-based training(21). Indeed, when designing a simulation-based scenario, a teaching plan that includes learning objectives is essential(6, 22). The decision to use low- or high-technology materials depends on local resources and the clinical setting(6). A high-fidelity simulator is an expensive technology for assessment and can support a real environment, so it has an important impact on trainees’ confidence for specific learning experiences, especially in critical situations but the cost of high-fidelity manikins limits its availability [1, 2].

In our study, we used a video-based facilitator-guided debriefing to enhance learners’ reflective thinking. Post-simulation debriefing is one of the most important components of simulation-based education and is very important for the learning experience(6). It is an important way to provide feedback on practice, allowing learners to reformulate the experienced scenario, discuss and learn from mistakes, and identify potential pitfalls (6, 23, 24). In a clinical trial, Gamboa et al. evaluated two debriefing strategies for developing neonatal resuscitation skills and found that there was no significant difference between oral or video-based debriefing and that both strategies increased participants’ behavioral and technical skills [25]. Therefore, determining the best method for debriefing is based on contexts and specific learning needs [6]. In a scoping review, Fawk et al. found that there are knowledge gaps in the use of briefing/debriefing in neonatal resuscitation, including its impact on short- and long-term clinical outcomes [26]. Neonatal resuscitation is a high-risk situation that can be very stressful for providers, so it seems unreasonable that one would allow junior residents to participate in neonatal resuscitation without being exposed to different scenarios in a simulation environment. In recent years, the limitation of working hours has led to a decrease in the experience of residents in emergency resuscitation of newborns. On the other hand, in recent years, the methods of optimal management of newborns have gradually changed towards non-invasive methods [6]. All these reasons prevent the acquisition and retention of skills [3, 27]. The results of the research have shown patterns of skill decline in pediatric residents. Therefore, NRP skills should be boosted by simulation-based mastery learning booster sessions in pediatric residents [18, 28].

Although the role of simulation-based training in increasing the knowledge and skills of trainees has been proven, the true effect of team training on clinical outcomes, neonatal mortality, and morbidity has not been revealed, and data on clinically relevant outcomes are scarce and further studies are needed [4, 6]. The resuscitation certificate for residents cannot be obtained immediately after the training courses and requires maintaining a high level of competence. Common barriers to implementing simulation-based training include time, organizational challenges, lack of support from policymakers, and cost [6, 29]. The cost of materials may be reduced through innovative solutions to use existing equipment from the clinical setting, such as the Help Baby Breath manikin we used, which is one of the positives of our study in a low-resource setting.

Our study has limitations: First, our study is not a randomized controlled trial and there are no comparisons with previous teaching methods (without team-based simulation) to demonstrate this method is superior. Second, while the simulation was run as a team, there was no specific teaching or evaluation on teamwork or other non-technical skills. The teams were physician-based with a single nurse actor, which may not reflect actual team compositions, and for logistical reasons, we were unable to assess level four outcomes (real-life impact) and did not assess patient outcomes in the delivery room. Third, we used a low-fidelity manikin due to the situation of our local context and our sample size was small.

## Conclusion

Our study highlighted that using low-cost local materials to create new training models can lead to success in team-based simulation training in the context of newborn resuscitation in low-resource countries. The results showed that team-based simulation training had a positive and significant increase in the knowledge, skill, and confidence of pediatric residents and abilities related to competence. Further studies with more sample sizes are recommended to evaluate its impact on real-life practice and especially its effect on the acquisition of team leadership skills in the delivery room (Kirkpatrick’s fourth level Results).

### Electronic supplementary material

Below is the link to the electronic supplementary material.

Supplementary Material 1

Supplementary Material 2

Supplementary Material 3

## Data Availability

The datasets used and/or analyzed during the current study are available from the corresponding author upon reasonable request.
