# Supplementary material for: Enhancing residents’ neonatal resuscitation competency through team-based simulation training: an intervention educational study
Source: BMC Med Educ. 2023 Oct 10;23:743. doi: 10.1186/s12909-023-04704-4 (PMC10563222; doi:10.1186/s12909-023-04704-4)
Supplement: Supplementary file 1 — Supplementary Material 1 [file 12909_2023_4704_MOESM1_ESM.docx]

| Learner: Date: Evaluator:  SCORING: 0= Not done 1= Done incorrectly, incompletely or out of order 2= Done correctly in order |
| --- |

| **Lesson** | **Item** | **0** | **1** | **2** |
| --- | --- | --- | --- | --- |
| 2 | **Asks 4 pre-birth questions (Expected GA, Fluid clear, UC management plan, Risk factors)** |  |  |  |
| 2 | **Discusses plan and assigns roles to team members** |  |  |  |
| 2 | **Checks Equipment inc. Bag, Mask & Oxygen Supply** |  |  |  |
| 2 | **Asks 3 assessment questions (Term, Tone, Breathing or Crying)** |  |  |  |
| 3 | **Warm dry stimulate and removes wet towels Position the airway and suction if necessary** |  |  |  |
| 3 | **Assesses respirations +/- heart rate**  Initiates monitoring for pulse oximeter probe to right wrist |  |  |  |
| 4 | **Indicates need for and initiates positive** **positive-pressure ventilation**(Apnea or gasping, heart rate**)** |  |  |  |
| 4 | Checks for rising heart rate after 15 seconds of PPV |  |  |  |
| 4 | Takes corrective action when heart rate not rising & chest not moving (Mask readjustment Reposition; Suction mouth & nose Open mouth; Pressure increase; Alternate airway) |  |  |  |
| 4 | **Provides effective positive pressure ventilation (40-60 bpm) for 30 seconds** |  |  |  |
| 4 | Re-evaluates heart rate**(Heart rate< 60 bpm)**  Consider intubation and apply ECG if not already done |  |  |  |
| 5 | Demonstrates correct technique for intubation or assisting with intubation |  |  |  |
| 6 | **Identifies need to start chest compressions**  (Increases oxygen to 100% , Demonstrates correct compression technique for 60 seconds (2-thumb method, compression depth 1/3 anterior-posterior diameter, complete recoil of chest) |  |  |  |
| 6 | Takes corrective action when heart rate not rising using the “CARDIO” mnemonic (Is the Chest moving with each breath; Is the Airway secured; Is the Rate of compressions to ventilations 3:1; Is the Depth of compressions 1/3 of the AP diameter; Is the Inspired Oxygen being delivered at 100%) |  |  |  |
| 7 | **Identifies need for epinephrine (Heart rate<60bpm)** |  |  |  |
| 7 | Identifies correct dose and route for epinephrine [0.02mg/kg IV (0.2mL/kg) and 0.1mg/kg (1mL/kg) ET |  |  |  |
| 7 | Administers ET dose while umbilical catheter being prepared , Prepares or assists with preparation of umbilical catheter for insertion Inserts or assists with insertion of umbilical venous catheter Administers epinephrine via umbilical venous catheter with 3 ml flush |  |  |  |
| 7 | **Identifies need for volume administration and administers correct solution, volume and rate of infusion** |  |  |  |
| 3 | Administers blended oxygen to meet targeted saturations using pulse oximeter during resuscitation |  |  |  |
| closure | sequence ,Continues/discontinues positive-pressure ventilation appropriately or weans oxygen correctly |  |  |  |

Performed all shaded items correctly? Yes □ No □ Re-evaluate **□**

Minimum passing SCORE is 32/38

**Supplementary 1.** *Neonatal Resuscitation Program Megacode Assessment (Individual Integrated Skills Station Assessment Form)*
