# Supplementary figures and images for: Enhancing residents’ neonatal resuscitation competency through team-based simulation training: an intervention educational study
Source: BMC Med Educ. 2023 Oct 10;23:743. doi: 10.1186/s12909-023-04704-4 (PMC10563222; doi:10.1186/s12909-023-04704-4)

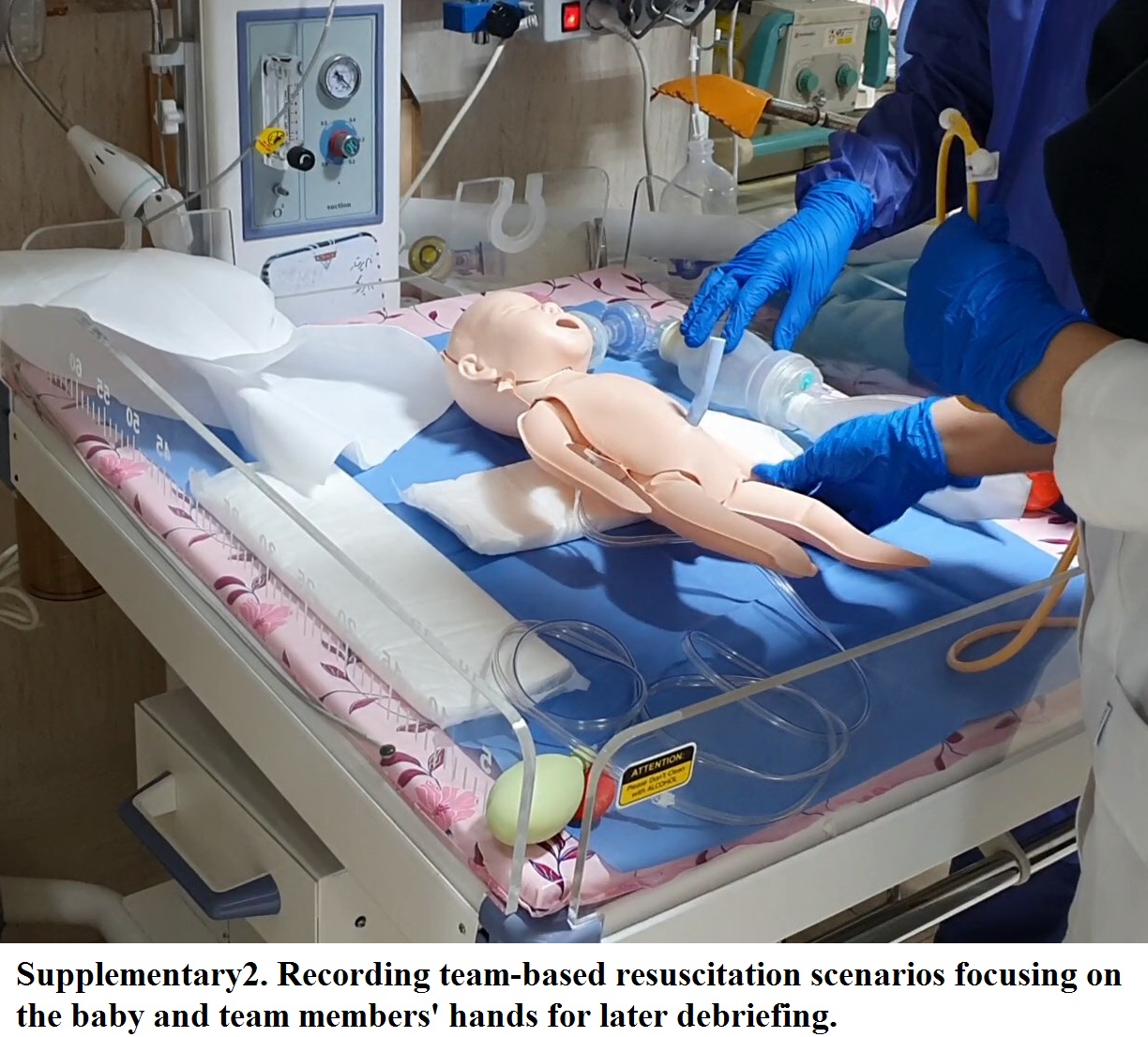

Supplement: Supplementary file 2 — Supplementary Material 2 [file 12909_2023_4704_MOESM2_ESM.jpg]
