# Supplementary material for: Enhancing residents’ neonatal resuscitation competency through team-based simulation training: an intervention educational study
Source: BMC Med Educ. 2023 Oct 10;23:743. doi: 10.1186/s12909-023-04704-4 (PMC10563222; doi:10.1186/s12909-023-04704-4)
Supplement: Supplementary file 3 — Supplementary Material 3 [file 12909_2023_4704_MOESM3_ESM.doc]

| Course: Neonatal resuscitation team-based simulation  Date of holding:  Instructor's(Professor) name: | | | | | |
| --- | --- | --- | --- | --- | --- |
| **Evaluation criteria** | **Weak**  **(1)** | **Medium**  **(2)** | **Good**  **(3)** | **Very good**  **(4)** | **Excellent**  **(5)** |
| **A- Content** |  | | | | |
| Applicability of the content (expression of evidence and practical scenarios) |  |  |  |  |  |
| Content up-to-date |  |  |  |  |  |
| Increasing your information about the training course |  |  |  |  |  |
| The potential created by this session for job activities |  |  |  |  |  |
| Suitability of program content to your expectations |  |  |  |  |  |
| The attractiveness of the course for continued attendance |  |  |  |  |  |
| **B-Professor ( Instructor)** |  | | | | |
| The ability to express and convey content |  |  |  |  |  |
| Using new methods of teaching and learning |  |  |  |  |  |
| The level of expertise and mastery of the instructor |  |  |  |  |  |
| Presenting ,progressing and redirecting the scenario |  |  |  |  |  |
| Introduction and presentation of teaching references |  |  |  |  |  |
| Creating motivation and attracting the participation of learners |  |  |  |  |  |
| Use of teaching instrument |  |  |  |  |  |
| Create interest in asking and answering |  |  |  |  |  |
| Ability to manage time |  |  |  |  |  |
| General evaluation of training course management |  |  |  |  |  |
| **C. Organization** |  | | | | |
| Course notification and planning coordination |  |  |  |  |  |
| Educational facilities and aids |  |  |  |  |  |
| The behavior of the presenters |  |  |  |  |  |
| Educational environment in terms of physical facilities (light, sound, comfort, etc.) |  |  |  |  |  |
| **..................................... Total scores** | | | | | |
| : Positive points of the course | | | | | |
| :Negative points of the course | | | | | |
| In your opinion, the content of this course will be effective in what part of your activities? | | | | | |
| What other jobs do you think this course is suitable for? | | | | | |

**Supplemantary3.** Survey form to evaluate overall satisfaction score.
